# Drivers, facilitators, and sources of stigma among Akha and Lahu hill tribe people who used methamphetamine in Thailand: a qualitative approach

**DOI:** 10.1186/s12889-022-13094-z

**Published:** 2022-04-02

**Authors:** Anusorn Udplong, Tawatchai Apidechkul, Peeradone Srichan, Thanatchaporn Mulikaburt, Pilasinee Wongnuch, Siwarak Kitchanapaibul, Panupong Upala, Chalitar Chomchoei, Fartima Yeemard, Ratipark Tamornpark, Onnalin Singkhorn

**Affiliations:** 1 School of Health Sciences, Mae Fah Luang University, 333 Moo 1, Ta Sud Subdistrict, Muang District, Chiang Rai, Chiang Rai Province 57100 Thailand; 2 Center of Excellence for Hill Tribe Health Research, Mae Fah Luang University, Chiang Rai, Thailand; 3 School of Nursing, Mae Fah Luang University, Chiang Rai, Thailand

**Keywords:** Akha, Lahu, Hill tribe, Thailand, Substance use, Methamphetamine, Stigma

## Abstract

**Background:**

The stigma related to drug use has several impacts, including effects on users’ physical and mental health. Methamphetamine is a major drug that is used among hill tribes living in the border areas of Thailand and Myanmar. This study aimed to understand the drivers, facilitators, sources and outcomes of the stigma surrounding drug use, including the expectations among Akha and Lau hill tribe people who use methamphetamine in Thailand.

**Methods:**

Qualitative data were used to elicit information from key informants and members of the hill tribes who used methamphetamine. The questionnaire was developed from a literature review and tested for validity before use. In-depth interviews were used to confidentially gather information from the participants in private rooms in villages. Each interview lasted 45 min, and a thematic analysis was conducted to examine the findings.

**Results:**

A total of 46 participants were recruited to provide information; 95.7% were male, and 50.0% were aged 15–34 years. The majority were married (47.8%), 76.1% were Christian, and 45.7% graduated high school. Six drivers of stigma were detected: being poor, illiterate, unemployed, working aged, female, and married. Culture and tribe acted as facilitators of the stigma attached to methamphetamine use. Four sources of stigma were found: self, family members, peers, and community members. Three outcomes of stigma were determined: poor physical health, mental health, and relationships with others. There were four levels of expectations: no expectations, expectations for themselves, expectations for their family members, and expectations for their community members.

**Conclusions:**

Many personal traits, people living nearby, and socioeconomic factors, including culture and tribes, act as drivers, facilitators, and sources of stigma among hill tribe people who use methamphetamine. A program to reduce methamphetamine use among hill tribes should be implemented, which could eventually minimize stigma.

**Supplementary Information:**

The online version contains supplementary material available at 10.1186/s12889-022-13094-z.

## Introduction

In 2020, the United Nations (UN) reported that 269 million people used drugs worldwide [1], and methamphetamine accounted for the greatest proportion of drugs used [2]. There are several impacts of methamphetamine use in both physical and mental aspects in all age categories [3–5]. There are several economic and social consequences of methamphetamine use, such as daily spending on pills and the loss of human life security for drug users [6]. Moreover, stigma is one of the major issues experienced by methamphetamine users [7]. The American Psychological Association (APA) defines stigma as an individual’s negative perception of some specific characteristics of themselves or others that has an impact on physical and mental health and personal and social relations [8]. Several individual characteristics have been clearly defined as sources of stigma, such as being a minority [9, 10], using different languages [11, 12], living in lower socioeconomic environments [13], and being a substance abuser [14–17]. Having multiple such traits, for instance, being both a minority and substance abuser, increases the impact of stigma. The World Health Organization (WHO) reported that stigma is the major cause of discrimination and exclusion, and it affects individual self-esteem and limits socialization [18]. Stigma is a major threat to mental health and human rights, especially among people who are already marginalized, such as members of the hill tribes and stateless populations in Thailand [18, 19].

Hill tribe people living in Thailand are a group of people who have migrated from South China in the past couple of centuries and settled on the border areas of Thailand and Myanmar [20]. In 2020, more than 4 million hill tribe people lived in Thailand [21, 22]. These people have their own language, culture, and lifestyle [22]. There are six major groups: Akha, Lahu, Hmong, Yao, Karen, and Lisu [23]. Akha and Lahu are the first and second largest populations among the hill tribes [23]. Even though they have been living in Thailand for a long time, approximately 30% of them have not been granted Thai citizenship, which would give them a 13-digit identification card [24] used to access all public services, including education and health care systems [24]. A large proportion (65%) of Akha and Lahu hill tribe people live below the national poverty level [24, 25] and have poor education [25] and low family income, which adversely affects quality of life and contributes to substance abuse. Hill tribe people without Thai identification cards (ID cards) were defined as the stateless population, regardless of how long these people lived in Thailand [26]. Compared to those who hold Thai ID cards, the stateless population had difficulty accessing all public services, including health care services and education systems [27, 28]. For people living in Thailand, including people living in the border areas of Thailand-Myanmar, people with poorer education have fewer choices to work in a high-compensation industry [29, 30].

The location of the hill tribe homes on the border areas of Thailand and Myanmar makes it easy for them to access illegal drugs, especially methamphetamines. Consequently, a large proportion of Akha and Lahu people aged 15–24 years use methamphetamine—14.3 and 14.8%, respectively [31, 32]. Methamphetamine use in this population has been classified according to different causes, such as loneliness [33, 34], personal and family relationship problems [34, 35], and persuasion by peers [36, 37]. In addition to one or more of these causes, the stigma of being a minority, living in poor socioeconomic conditions and using methamphetamine has a tremendous impact on their daily lives.

Therefore, this study aimed to understand the drivers, facilitators, sources of stigma, and outcomes, as well as the expectations among Akha and Lahu hill tribe people using methamphetamine.

## Methods

A qualitative approach was used to elicit information from key informants. The key informants were the Akha and Lahu hill tribe people aged 15 years and older using methamphetamines and living in the hill tribe villages located at the border of Thailand and Myanmar in Chiang Rai Province, Thailand.

Questions to guide the interviews were developed from a literature review [38], including information from a preliminary discussion with hill tribe village headmen. There were nine questions: 1) Do you use any drugs, such as methamphetamines? 2) How long have you used methamphetamines? 3) What did your family think or how did they respond to your use of methamphetamines? 4) What did your friends think or how did they respond to your use of methamphetamines? 5) What did your community members think or how did they respond to your use of methamphetamines? 6) How did you feel about the responses from your family members, friends, and community members? 7) How did you respond to their actions? 8) How do you feel about yourself as a methamphetamine user? 9) What do you expect from family members, friends, and community members regarding your use of methamphetamines? (Additional file 1. Question guide).

The validity and reliability of the questions were assessed. Three experts (one stigma study expert from Mae Fah Luang University, one public health professional who worked in the hill tribe community, and one clinician who worked in the drug session clinic at Chiang Rai Regional Hospital) were invited to comment on the validity of the questions and the content of the study. The comments were carefully considered and improved before moving to the next step. The questions were also piloted in five hill tribes with similar characteristics in Mae Chan District, Chiang Rai, Thailand. Finally, the questions were refined by the research team. Eleven researchers on this project worked at a university and had various backgrounds: four people were qualitative-based, three people were public health and epidemiology specialists, two were in the social and behavioral sciences, and two were psychologists. Five members of the researcher team (three males and two females) who held PhDs in qualitative research and were familiar with the hill tribes were assigned to be interviewers. All researchers had 4–14 years of experience in researching these populations.

Appointments for each interview were made 5 days in advance. A village headman was contacted and provided preliminary characteristics of prospective participants. On the day of the interview, a participant was questioned according to the study criteria, especially methamphetamine use and experiences with stigma, before the interview. Most of the methamphetamine users in the village were located by the snowball technique beginning with requests to government agencies for potential subjects. Almost all the methamphetamine users in the hill tribe villages were recruited into the methamphetamine secession program, which was operated by the Ministry of Interior [39]. Then, someone who passed the program and was recruited into the program was the best option to recruit the remaining participants. Those who passed the program were well known by the community members because the program was operated by the community members.

Those who met the criteria were asked to provide consent on a voluntary basis before the interview. For those aged less than 18 years, informed consent was also obtained from their parents. An interview was conducted in a private and confidential room. In the initial stage of the interview, greetings and conversations were used to make the interviewee and interviewer familiar with one another. Interviewees were provided information regarding the interviewer before details of the guide were given. The interview was gender-matched between the interviewer and interviewee. All interviews were recorded, and field notes were taken after receiving approval from the interviewee. Interviews lasted 45 min (average of 43.3 min).

After completion of the interviews, all recordings were transcribed. The transcripts were checked for errors and missing information before being returned to the participants who owned the story. Intercoder reliability was used to ensure the quality of the codes obtained before all researchers read the transcripts before coding, and coding trees were developed. The data were transferred into the NVivo program (NVivo, qualitative data analysis software; QSR International Pty Ltd., version 11, 2015) for thematic analysis. The final analysis was conducted by researchers who organized, compared, and contrasted codes to define the key themes. Their findings were reviewed by two external experts who had backgrounds in stigma research related to substance abuse.

All research tools and procedures were approved by the Chiang Rai Public Health Human Research Ethics Committee (reference No 69/2564). All documents were destroyed properly after the research was completed.

## Results

### General characteristics

A total of 46 participants provided information for the study; 95.7% were male, and 50.0% were aged 15–34 years. The majority were married (47.8%), 45.7% graduated high school, 52.2% were Lahu, and 76.1% were Christian. Almost half of the participants were employed (47.8%), 39.2% had incomes ≤4999 baht per month, and 89.1% held Thai ID cards (Table 1).

**Table 1:** Characteristics of participants

Characteristic	Akha n (%)	Lahu n (%)	Total n (%)
**Sex**			
Male	20 (43.5)	24 (52.2)	44 (95.7)
Female	2 (4.3)	0 (0.0)	2 (4.3)
**Age** (years)			
15–34	6 (13.0)	17 (37.0)	23 (50.0)
35–59	14 (30.4)	7 (15.2)	21 (45.7)
≥ 60	2 (4.3)	0 (0.0)	2 (4.3)
mean = 34.2, SD = 12.5 min = 15, max = 60			
**Marital status**			
Single	4 (8.7)	13 (28.3)	17 (37.0)
Married	14 (30.4)	8 (17.4)	22 (47.8)
Previously married	4 (8.7)	3 (6.5)	7 (15.2)
**Education**			
Noneducated	15 (32.6)	1 (2.2)	16 (34.8)
Primary school	1 (2.2)	7 (15.2)	8 (17.4)
High school	5 (10.9)	16 (34.8)	21 (45.7)
University	1 (2.2)	0 (0.0)	1 (2.2)
**Religion**			
Buddhist	1 (2.2)	10 (21.7)	11 (23.9)
Christian	21 (45.7)	14 (30.4)	35 (76.1)
**Occupation**			
Unemployed	5 (10.9)	1 (2.2)	6 (13.1)
Employed	9 (19.6)	13 (28.2)	22 (47.8)
Farmer	8 (17.4)	8 (17.4)	16 (34.8)
Student	0 (0.0)	2 (4.3)	2 (4.3)
**Income** (baht per month)			
No income	4 (8.7)	3 (6.5)	7 (15.2)
≤ 4999	10 (21.8)	8 (17.4)	18 (39.2)
5000–9999	6 (13.0)	9 (19.6)	15 (32.6)
≥ 10,000	2 (4.3)	4 (8.7)	6 (13.0)
**Has a Thai ID card**			
Yes	18 (39.1)	23 (50.0)	41 (89.1)
No (Stateless people)	4 (8.7)	1 (2.2)	5 (10.9)
**Previously**			
Yes	9 (19.6)	10 (21.7)	19 (41.3)
No	13 (28.3)	14 (30.4)	27 (58.7)

### Characteristics of the stigma

Among the Akha and Lahu hill tribe people who used methamphetamines and experienced stigma, drivers, facilitators, and sources of stigma and health outcomes were as follows.

#### Drivers

Six factors were detected as drivers of stigma among the hill tribe members who used methamphetamines: poverty, illiteracy, unemployment, working age, sex, and marital status.

##### Poor people

Poor people who used methamphetamine were likely to encounter stigma. Those who used methamphetamine and lived in high-income families suffered less from stigma than those who lived in poor families. One interesting reason for methamphetamine use among the hill tribe members was to have energy for daily work, especially farming. This scenario was presented by impoverished people, and they reported much higher stigma than those living in better socioeconomic environments.

A 40-year-old man stated the following [P#16]:

“I am 40 years old and responsible for my family. As a family leader, I need to earn money for my family, and I have to work hard to get money. I need methamphetamines to have energy for work, but I feel bad when people look down on me.”

A 52-year-old man stated the following [P#32]:

“I am a poor farmer and use methamphetamines. Many of my friends also use methamphetamines, but poor people are treated badly by the people in the village. I don’t understand why they blame only poor people while the rich people who use methamphetamines do not have these bad experiences.”

A 21-year-old man stated the following [P#8]:

“Because my family is poor, I need to work very hard to earn money. Then, I decided to use methamphetamines a couple years ago. Since then, my friends look at me with unfriendly eyes. I have realized that if we are poor and use methamphetamines, we will get a lot of bad signs from people living around us.”

Family income was one of the drivers of the stigma among the Akha and Lahu hill tribe people who used methamphetamines.

##### Illiteracy

A large proportion of participants were illiterate. Those who were illiterate experienced stronger negative experiences with stigma than those who had some level of education or were in school.

A 45-year-old man stated the following [P#1]:

“My uncle was angry with me when he learned I was using methamphetamines. He said that if I attended school, I might not use methamphetamines. I think, education is not truly linked to being a bad or a good person at all. My friends who use drugs, many of whom attend school, aren’t blamed. However, I feel that people that have some level of schooling show a little negative behavior toward me.”

A 28-year-old man stated the following [P#12]:

“I didn’t attend school because I didn’t want to. In addition, yes, I use drugs now. People in my village usually think I am a junkie. They put me in a group of bad people who left school and used drugs. In their minds, drug users always do bad things such as stealing things in the village. I want to tell them that I am not a thief. I am just a drug user.”

A 52-year-old man stated the following [P#11]:

“I did not attend a school and have used methamphetamines for many years. I have also been arrested once. I know that being poor and using methamphetamines meant that I would not get any respect from people.”

Therefore, poor education was taken as a driver of the stigma experience among the Akha and Lahu hill tribe members who used methamphetamines.

##### Unemployment

Six people who were unemployed and used methamphetamines experienced a stronger stigma than those who had been employed or were currently working. Most of the Akha and Lahu hill tribe people have low family incomes, and almost all family members were taught to be hard working persons. Therefore, being unemployed was treated with stronger stigma from people in the drug users’ families and communities.

A 60-year-old man stated the following [P#46]:

“I used to be a farmer, but now I do not work due to my health problems. People look at me as a junkie who does not work and lives for drug use. I think it’s unfair to me.”

A 52-year-old man stated the following [P#11]:

“Now, my status is unemployed due to COVID-19. As I used drugs previously, my relatives perceive that I am a lazy person like other drug users.”

Unemployment was considered a significant driver of stigma among the Akha and Lahu hill tribe people who used methamphetamines.

##### Working age

Those aged 20–45 years who used methamphetamine faced a stronger stigma than younger or older persons. Commonly, the Akha and Lahu working-age members were expected to work very hard to support their families. Individuals of working age who used methamphetamines experienced a stronger stigma than younger or older people.

A 31-year-old man stated the following [P#37]:

“This year, I am 31 years old. In my culture, we believe that it’s almost a half of life, and I still suffer from the labeling of a drug user. My brother told me that I was not a young boy anymore and asked why I can’t quit drugs and make money to support my family. He compared me with my nephew, saying if I was a teenager, he could accept it. His words hurt me a lot.”

A 42-year-old man stated the following [P#36]:

“My friends said, ‘At your age, you can be a granddad, but you still make yourself a fool by using drugs.’ This made me very upset. I do not want anyone to judge me. I also don’t like when people compare me to the others, especially to the younger people.”

A 35-year-old man stated the following [P#20]:

“I used drugs for years, and I have gotten bad words from my father that a good person should work and support his family. I am just thinking about one of my peers. He is just 16 years old. He did not get any negative experiences from his father.”

A 30-year-old man stated the following [P#27]:

“I feel that those who use drugs and have a family are blamed more than those who do not have families and are younger. I have two kids, and my father always says that I have to work hard to support my family. I hear these words almost every day. I really do not like it.”

Because of the economic constraints among the Akah and Lahu hill tribe families, all working-age persons were expected to work very hard to support their families. Anyone who used methamphetamines experienced a larger impact of stigma from the people around them.

##### Female sex

Females who used methamphetamines experienced more stigma than males. Females were expected to be better persons than males. Using methamphetamines was judged as a bad lifestyle among the hill tribes. Female methamphetamine users were more likely to experience stigma than males.

A 42-year-old woman stated the following [P#38]:

“Being a female, I’m expected to be a good person in our culture. A good wife or mom should not use drugs. I have been labeled as a drug user by people, including my family members. I feel very bad.”

A 21-year-old woman stated the following [P#29]:

“I always hear people in my village gossip about me as a bad person, “a drug-addicted person,” which hurts me very much. To be honest, even though I use drugs, I’ve never done anything bad. I feel that I have gotten negative signs from people more than male drug users.”

Even though methamphetamine use was not accepted by the Akha and Lahu hill tribes and stigmatized, females who used methamphetamines were more impacted by stigma than males.

##### Marital status

Married individuals who used methamphetamines experienced more stigma than those who were single. The people in the village expected to see married people handle their familial roles and be able to support their families in terms of economic and other common roles. Therefore, those who were married and used methamphetamines were considered nonresponders to their roles. The stigma of their drug use could extend to their children.

A 28-year-old man stated the following [P#18]:

“I married 4 years ago. I use drugs sometimes while having stress. My family had high expectations of me, that I would be a good family man. I think that using drugs doesn’t mean I am a bad person. Even though I use drugs, I still work hard to support my family. However, do you believe it? I have gotten a lot of negative things from my family and people in this village.”

The stigma among those who used methamphetamines and were married came from the expectations of people living in the community that they should support their families. The impact of the stigma could extend to their children as well.

#### Facilitators

Culture and tribe were detected as facilitators of stigma faced by methamphetamine users. Some cultures in some tribes acted as facilitators, but others did not. In the tribes, methamphetamine users suffered from stigma at different levels.

##### Culture

Akha and Lahu cultures accepted opium use among older people, while the other tribe cultures did not. Acceptance of opium use has less impact on stigma than methamphetamine use because opium use, including opium use to relieve illness, has been integrated into the hill tribe lifestyle for a long time.

A 51-year-old man stated the following [P#30]:

“I had seen older people in my village using opium when I was young. It is normal to us to relieve pain from farming. In the Akha culture, we do not care if older people use opium. However, for young people, it is not good.”

A 38-year-old man stated the following [P#40]:

“I live in Lahu Village. We are okay with older people using opium. Sometimes they need to relax. However, methamphetamine use among younger people is not acceptable. After individuals use methamphetamines, they can do many bad things, including steal things of others. People do not accept the use of methamphetamines in the Lahu community. However, I personally think that the acceptance of using methamphetamines in Lahu is better than in other tribes like Lisu. The Lisu, they do not like anyone using methamphetamines.”

Akha and Lahu people who used methamphetamines experienced less stigma than the members of other tribes due to the impact of their culture on the acceptance of substance use.

##### Tribe

Comparing the tribes, some tribes viewed other tribes as belonging to lower social classes. For instance, the Lisu people viewed the Akha people as lower class based on their economic status and personal hygiene. Then, if the Akha people used methamphetamines, they experienced much more serious stigma from people who were in other tribes.

A 48-year-old stated the following [P#41]:

“I know many people who are Akha and Lisu. I feel that the Lisu people look down on us a lot. Many times, they said that we are just the laborers for them. If the Lahu people use drugs, they are blamed by the Lisu people. I know that it is not easy to change the perspective of Lisu people.”

The tribe was determined to be one of the facilitators of stigma among the Akha and Lahu hill tribe people who used methamphetamines.

#### Stigma making

In this study, we aimed to understand the stigma of using methamphetamines among hill tribe people. Then, methamphetamine use was the stigma maker.

#### Sources of stigma

Four sources of stigma were detected among the Akha and Lahu hill tribe people who used methamphetamines: self, family members, peers, and community members.

##### Self-stigma/self-blame

Among the Akha and Lahu hill tribe methamphetamine users, self-stigma was widespread. The source of self-stigma could be understood as poor self-esteem, feeling wrong about using, and feeling like a bad person. It was a common teaching among the hill tribe people that those who used drugs were bad people. Therefore, methamphetamine users were fully conscious of this teaching, felt they were bad people and blamed themselves. Once they felt they were bad people, they started to use drugs again.

A 45-year-old man stated the following [P#7]:

“I am the oldest son in my family, so my parents had high expectations for me. When they knew that I was using drugs, I felt I had been turned into the youngest son in my family. They do not respect me anymore. I feel disappointed in myself, but I really don’t know what to do next. I am a weak person who has failed in every way. I should not use drugs, but it is too late.”

A 23-year-old man stated the following [P#21]:

“I have been using drugs since I was 14 years old. My close friend convinced me to use methamphetamines. If I said no, I would be ignored by him and not be his friend. It was the first time I used drugs. If I could turn the time back, I would not have done it. It makes my life a failure. Today, I have not had the confidence to do anything. I do not dare go outside or talk to people. I make my mom cry.”

Methamphetamine use is a cause of self-stigma, and negative feelings cause its repeated use.

##### Stigma produced by family members

The stigma experiences among Akha and Lahu hill tribe methamphetamine users came in various forms, especially from family members. Parents spoke to their children, urging them to stop using methamphetamines. The repeated messages from parents regarding methamphetamine use was a pattern of stigma. Unfriendly words from spouses were another form of stigma. In severe cases, users were ignored by family members, physically abused, and asked to get out of the house.

A 15-year-old male stated the following [P#10]:

“My dad said that he does not like me, and I should not be his son. This sentence hurts me a lot. While my mom knew that I used the drug, she forced me to quit it. I told her I have tried many times, and I need understanding and encouragement from my family, but they don’t understand. They always say, ‘you are the mistake of the family.’ I cry every night.”

A 30-year-old man stated the following [P#22]:

“I have been married for 8 years and have one daughter. I and my wife often have arguments. She shouted at me that I was a pimp man who stripped her of money. In addition, she kicked me out of my home many times. These things make me feel like I am a bad and useless person.”

Family members created a significant amount of stigma by verbally and physically abusing the drug user.

##### Stigma produced by peers

Stigma among methamphetamine users was also generated by their peers. Most of the methamphetamine users reported that they were ignored by their peers and not invited to join common activities. A large number experienced an absence of peers in their lives while using methamphetamines.

An 18-year-old man stated the following [P#25]:

“I had many friends in the village previously. Some of them use drugs, and the rest don’t. I notice that my friends who do not use the drugs ignore me and try not to contact me or be friends with me anymore. In the past, we played football together, but since they learned that I have been using drugs, they never ask me to play with them anymore.”

A 42-year-old woman stated the following [P#38]:

“In our village, we have a group of housewives, and I am a member of the group. My friends try not to speak to me or contact me. They said that they didn’t want to be connected to a person like me. They are afraid that the people outside the group might think negatively of the group if I am a member. I feel lonely and upset.”

A 20-year-old man stated the following [P#25]:

“Before I used drugs, I had many friends. Once people knew that I was using a drug, most of my friends stopped contacting me. Sometimes, they speak very harsh words to me. I feel that I am living alone now.”

The stigma from their peers was presented as the absence of friends in their lives and a lack of invitations to participate in activities.

##### Stigma produced by community members

Methamphetamine users experienced gossip from the people in the community. Many users were treated as untrustworthy persons due to methamphetamine use and the suspicion that they would steal things from others.

A 30-year-old stated the following [P#22]:

“When things in the village disappeared, the villagers always accused me as the person who stole them. For example, a chicken of my neighbor’s was lost, and he accused me as the person who took the chicken. I have tried to explain that I did not do that, but nothing improved. He perceived that a person who uses drugs must be a bad person. Sometimes, I feel that it is not fair to me.”

A 48-year-old stated the following [P#34]:

“I have heard that people gossip about me using drugs. They sometimes use their body language to make me feel like a monster, such as using their eyes to tell their children not to get close to me. One time, we had a new house ceremony, and the host asked me not to join, as he did not want the majority to feel bad, but they did not care about my feelings.”

A 25-year-old stated the following [P#44]:

“Do you know, whenever bad things happened in our village, they always looked at me. Anything lost by anyone, people always looked at me, even when I said that I did not take it. However, nobody believes me.”

The methamphetamine users experienced mild to severe impacts from the stigma generated by community members. The impact of the stigma led to much suffering.

#### Outcomes

Three outcomes were detected as a result of the impact of stigma among the methamphetamine users: poor physical health, poor mental health, and poor relationships with others.

##### Poor physical health

The methamphetamine users’ experience of stigma reduced their appetites, resulting in weight loss. Some kept to themselves for long periods of time, and many health problems developed, such as stomach and joint pain.

##### Poor mental health

Most of the methamphetamine users faced mental health problems due to the effects of drugs and faced subsequent personality changes. Seven reported that they contemplated suicide, and six were required to visit a doctor. Moreover, a few individuals reported being caught trying to commit suicide and being transferred to a mental hospital.

##### Poor relationships with others

Almost all reported that they had poor relationships with family and community members. Most methamphetamine users were ignored by the people living around them.

### Expectations

There were four categories of expectations among the methamphetamine users: no expectations, their own expectations, expectations of family members, and expectations of community members.

#### No expectations

Six reported that they did not have any expectations of their lives. They just lived life day by day in the same routine. They did not need anything more and accepted everything that would happen in the future.

#### Expectations of themselves

Twenty-two users reported that they needed to stop using drugs and become normal members of the family and community. They needed to work and earn money for their lives and for their families. Three said they needed to go back to school and have a normal life like their friends.

#### Expectations of family members

Ten need to return to their families and live with their wives and children. Many said that they hoped to return to their families and live with their parents because their parents were getting old and had nobody to care for them.

#### Expectations of community members

Almost all Akha and Lahu hill tribe methamphetamine users reported that they needed everyone to listen to them and understand their situations. They hoped to return to their usual selves and have everyone treat them as members of the tribe and talk to them.

## Discussion

The stigma among the Akha and Lahu people who use methamphetamines and live in Thailand was presented as drivers, facilitators, sources, and outcomes. Being poor, illiterate, unemployed, working aged, female, and married were detected as drivers of stigma among the methamphetamine users. Some cultures and tribes were presented as facilitators of stigma among drug users. Four sources of stigma were found: self, family members, peers, and community members. Three significant outcomes were stimulated by stigma among Akha and Lahu hill tribe methamphetamine users: poor physical health, poor mental health and poor relationships with others. Regarding expectations, four different categories were detected: no expectations, expectations of themselves, expectations of family members, and expectations of community members.

Fewer female participants were recruited into the study than male participants in both the Akha and Lahu tribes. This coincides with a study conducted among Akha and Lahu youth on methamphetamine use in northern Thailand [31], which reported that 3.2% females and 27.0% males used methamphetamines. Several studies conducted in different countries supported the higher proportion of substance use, especially methamphetamine, in males than in females [40–42].

Several personal traits were found to be drivers of the stigma among Akha and Lahu hill tribe methamphetamine users. Low family economic status was a significant driver of stigma, as was illiteracy. This implies that those with lower socioeconomic status (SES) experience more severe stigma than those with high SES. This was supported by a study conducted by Jonhson et al. [43], which reported that those who used drugs and had high SES were less stigmatized than those who had high SES. Thus, it was possible that tribe members with poor SES who also used methamphetamines experienced a large impact from the associated stigma. Moreover, being female, married, and of working age acted as drivers of the stigma. These findings reflect the disproportionate impact of stigma among individuals with low SES and those of working age who were expected to work hard for their families’ well-being. Once people used methamphetamines, they were impacted by the stigma. In the hill tribe culture, particularly in the Akha and Lahu tribes, females were treated as second to males, and they performed all the housework on the farms [24, 33]. It was not acceptable for females to use substances, such as methamphetamines [44]. A study in Germany [45] supported that being female and younger were significant drivers of the stigma attached to substance use.

In our study, some tribes and some cultures were detected as facilitators of stigma. Social class exists among the hill tribes in Thailand [46]. Some tribes look at other tribes as second-class, such as the Lisu, who believe they are superior to the Akha [46]. This judgment is made based on economic status. Yunnan-Chinese views the Akha and Lahu [47] as workers on their farms. Thus, if methamphetamine users are the Yunnan-Chinese or Lisu people, they might not experience as strong an impact from the stigma as the Akha or the Lahu. In addition, the Lisu do not accept substance use in their culture. Then, the Lisu people have less opportunity to get the impact from the stigma caused by methamphetamine use in daily life.

Four sources of stigma were detected among Akha and Lahu methamphetamine users: self, family members, peers, and community members. This agrees with findings from a study conducted in the United States that reported a stigma attached to drug users, especially those who had specific characteristics, such as being black or having low SES [48]. Another study conducted in the United States reported that stigma from policy-makers and health care providers could impact the entire process of treatment, including resource allocation for the treatment of substance use disorders [49]. It was confirmed that being an Akha and Lahu hill tribe member and being poor were the sources of stigma among the methamphetamine users in this study.

There were several health outcomes detected from the stigma among the hill tribe methamphetamine users: poor physical health and poor mental health. Several studies have reported the negative impact of methamphetamine use on physical and mental health. For example, Darke et al. [50], Stuart et al. [51]**,** Zwick et al. [52], and Crapanzano et al. [53] reported that among substance users, stigma had a great adverse impact on their health. Wogen et al. [17] reported that the stigma experienced among substance users had a negative impact on accessing mental health clinics and became a severe mental health problem. Gutierrez et al. [54] reported that those who used substances and experienced stigma were less likely to seek medical care and eventually had poor health. Our study confirmed that methamphetamine users among the Akha and Lahu people experienced damaging stigma.

There were four aspects of the Akha and Lahu hill tribe methamphetamine users’ expectations: no expectations, expectations of themselves, expectations of their families, and expectations of community members. The stigma faced by the methamphetamine users was of their own making and incurred by people living around them. The major expectation of the participants in our study was that they needed an understanding of the people around them. However, a few participants reported that they had no expectations, which might indicate that they did not plan to stop using drugs. A large proportion reported that they had hope of making their lives better, which was presented in the form of self-expectations. Many people also reported that they needed understanding from family and community members, especially the opportunity to become normal people like those who did not use drugs. There is no scientific report available on this issue. However, this paper clearly showed that Akha and Lahu people who used methamphetamines need help and opportunities to deal with the problems in their lives. This issue requires further study before it can be used to develop proper guidelines to help these populations.

A few limitations were found during this study. First, a small number of female participants were recruited. This is because a small proportion of Akha and Lahu females use methamphetamine. However, most of the information obtained for our study comes from Akha and Lahu males. Additionally, recruiting participants was slightly difficult because methamphetamine use is illegal in Thailand. Seven potential recruits refused to provide information. This might impact the study’s findings.

## Conclusion

The hill tribe people face the impact of the stigma associated with their methamphetamine use. Several individual characteristics, including socioeconomic status, culture and being a tribe member, act as stigma drivers and facilitators. The behavior of methamphetamine users is the stigma maker. There are four sources of stigma: self, family members, peers, and community members. People who use methamphetamine face three possible outcomes from the stigma: poor physical health, mental health, and relationships with others. Moreover, there are four levels of expectations among the hill tribe members who use methamphetamine: no expectations/nothing needed in their lives; the expectation that they could have a better life with a good job and earn more money; the expectation that family members will understand them and that they can live together; and the expectation that community members will see them as normal people.

There is a stigma attached to being a methamphetamine user among the hill tribe people. Therefore, implementing a reduction in the use of methamphetamine could minimize the impact of the stigma on users and their families. Holistic and inclusive approaches constructed based on cultures and socioeconomic status, should be applied to address the problem.

## Supplementary Information

**Additional file 1.** Question guide.

## Data Availability

The datasets generated and analyzed during the current study are not publicly available due to the transcripts containing full raw data that could be reflected to a specific participant, but are available from the corresponding author on reasonable request.

## Abbreviations

APA: American Psychological Association
SES: Socioeconomic status
UN: United Nations
WHO: World Health Organization
