# Supplementary material for: Drivers, facilitators, and sources of stigma among Akha and Lahu hill tribe people who used methamphetamine in Thailand: a qualitative approach
Source: BMC Public Health. 2022 Apr 2;22:642. doi: 10.1186/s12889-022-13094-z (PMC8976508; doi:10.1186/s12889-022-13094-z)
Supplement: Supplementary file 1 — Additional file 1. Question guide. [file 12889_2022_13094_MOESM1_ESM.docx]

**Question guide**

1) Do you use any drug, such as methamphetamines?

2) How long have you used methamphetamines?

3) What did your family think or how did they respond to your use of methamphetamines?

4) What did your friends think or how did they respond to your use of methamphetamines?

5) What did your community members think or how did they respond to your use of methamphetamines?

6) How did you feel about the responses from your family members, friends, and community members?

7) How did you respond to their actions?

8) How do you feel about yourself as a methamphetamine user?

9) What do you expect from family members, friends, and community members regarding your use of methamphetamines?
